# Tetra­kis(2,2′-bipyrid­ine)di-μ_3_-hydroxido-bis(μ-2-oxidobenzoato)tetra­copper(II) dinitrate tetra­hydrate

**DOI:** 10.1107/S1600536811011433

**Published:** 2011-04-07

**Authors:** Miao Feng, Chao Gu, Huai-Feng Mi, Tong-Liang Hu

**Affiliations:** a Biochemical Section of Key Laboratory of Functional Polymer Materials, Ministry of Education of China, Chemical School of Nankai University, 300071 Tianjin, People’s Republic of China; b Department of Chemistry, Nankai University, 300071 Tianjin , People’s Republic of China

## Abstract

The tetra­nuclear title complex, [Cu_4_(C_7_H_4_O_3_)_2_(OH)_2_(C_10_H_8_N_2_)_4_](NO_3_)_2_·4H_2_O, has a crystallographically imposed centre of symmetry. The Cu^II^ atoms display a distorted square-pyramidal coordination geometry and are linked by two μ_2_-phenolate O atoms from the salicylate ligands and two μ_3_-hydroxo groups, forming a Cu_4_O_4_ core that adopts a ‘stepped-cubane’ geometry. In the crystal, the cations are linked by O—H⋯O hydrogen bonds to the nitrate anions, which are in turn connected *via* O—H⋯O inter­actions to centrosymmentric water tetra­mers.

## Related literature

For the structures of related complexes, see: Albada *et al.* (2002 ▶); Chandrasekhar *et al.* (2000 ▶); Lu *et al.* (2007 ▶); Sletten *et al.* (1990 ▶); Zheng & Lin (2002 ▶); Fan *et al.* (2009 ▶); Li *et al.* (2008 ▶).
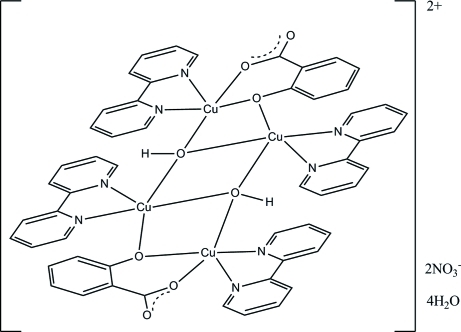


## Experimental

### 

#### Crystal data

[Cu_4_(C_7_H_4_O_3_)_2_(OH)_2_(C_10_H_8_N_2_)_4_](NO_3_)_2_·4H_2_O
*M* *_r_* = 1381.20
Triclinic, 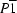

*a* = 10.280 (2) Å
*b* = 11.777 (2) Å
*c* = 12.276 (3) Å
α = 113.66 (3)°
β = 95.19 (3)°
γ = 96.58 (3)°
*V* = 1337.0 (5) Å^3^
*Z* = 1
Mo *K*α radiation
μ = 1.66 mm^−1^
*T* = 113 K
0.22 × 0.06 × 0.02 mm

#### Data collection

Rigaku Saturn70 diffractometer
Absorption correction: multi-scan (*CrystalClear*; Rigaku, 2005 ▶) *T*_min_ = 0.870, *T*_max_ = 1.000
17013 measured reflections
6339 independent reflections
4704 reflections with *I* > 2σ(*I*)
*R*_int_ = 0.049

#### Refinement

*R*[*F* ^2^ > 2σ(*F* ^2^)] = 0.040
*wR*(*F* ^2^) = 0.086
*S* = 1.04
6339 reflections
388 parameters
H-atom parameters constrained
Δρ_max_ = 0.81 e Å^−3^
Δρ_min_ = −0.55 e Å^−3^

### 

Data collection: *CrystalClear* (Rigaku, 2005 ▶); cell refinement: *CrystalClear*; data reduction: *CrystalClear*; program(s) used to solve structure: *SHELXS97* (Sheldrick, 2008 ▶); program(s) used to refine structure: *SHELXL97* (Sheldrick, 2008 ▶); molecular graphics: *SHELXTL* (Sheldrick, 2008 ▶); software used to prepare material for publication: *publCIF* (Westrip, 2010 ▶).

## Supplementary Material

Crystal structure: contains datablocks I, global. DOI: 10.1107/S1600536811011433/gk2359sup1.cif

Structure factors: contains datablocks I. DOI: 10.1107/S1600536811011433/gk2359Isup2.hkl

Additional supplementary materials:  crystallographic information; 3D view; checkCIF report

## Figures and Tables

**Table 1:** Hydrogen-bond geometry (Å, °)

*D*—H⋯*A*	*D*—H	H⋯*A*	*D*⋯*A*	*D*—H⋯*A*
O8—H1*W*⋯O6^i^	0.79	2.18	2.939 (3)	163
O8—H2*W*⋯O9^ii^	0.88	2.00	2.845 (3)	163
O9—H3*W*⋯O8^iii^	0.74	2.04	2.745 (3)	160
O4—H4*W*⋯O7	0.73	2.13	2.838 (3)	164
O9—H5*W*⋯O2^iv^	0.78	2.02	2.791 (3)	169

Symmetry codes: (i) 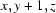; (ii) 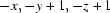; (iii) 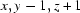; (iv) 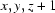.

**Fig. 1.**
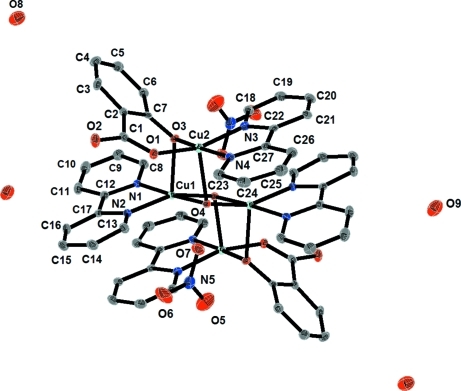
The structure of the title compound with displacement ellipsoids drawn at the 50% probability level. Unlabeled atoms are related to the labeled ones by the symmetry operation 1-x, 1- y, 1-z.

**Fig. 2.**
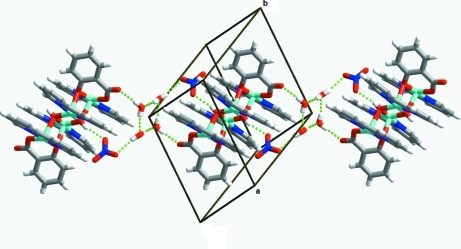
A packing diagram of the title compound. The O—H···O hydrogen bonds are shown as dashed lines.

## supplementary crystallographic information

### Comment

Recently, some tetranuclear hydroxo-bridged copper(II) complexes with cubane and the chair-like structure have been reported (Zheng & Lin, 2002; Sletten *et al.*, 1990; Albada *et al.*, 2002; Lu *et al.*, 2007; Chandrasekhar *et al.*, 2000; Fan *et al.* 2009; Li *et al.* 2008). In this paper, the crystal structure of a new copper(II) complex exhibiting a chair-like tetranuclear motif is presented.

The atom-numbering scheme of the title compound is shown in Fig. 1. The title complex has a crystallographically imposed centre of symmetry, and consists of a chair-like [Cu_4_(C_7_H_4_O_3_)_2_(OH)_2_(bpy)_4_]2^+^ dication (bpy = 2,2'-bipyridine), two nitrate anions, and four lattice water molecules. The coordination geometry around each copper(II) ion can be described as a five-coordinate distorted square pyramid. In the crystal packing, the nitrate counter-anions stabilize the crystal structure through water O—H···O nitrate hydrogen bonds and the complex molecules are linked into one-dimensional chains by intermolecular O—H···O bonding interactions involving the solvent water molecules and the nitrate counter-anions (Fig. 2 and Table 1).

### Experimental

A mixture of salicylic acid (0.05 mmol), copper nitrate trihydrate (0.05 mmol), 2,2'-bipyridine (0.05 mmol) and 10 ml H_2_O were put into a 23-ml Teflon lined reactor and heated at 418 K in oven for 48 h. After the autoclave was cooled during 24 h to room temperature, the solid was filtered off. The resulting filtrate was allowed to stand at room temperature, and slow evaporation for 3 weeks afforded block single crystals.

### Refinement

H atoms bound to C atoms were positioned geometrically (C—H = 0.93 Å) and allowed to ride on their parent atoms with *U*_iso_(H) = 1.2*U*_eq_(C). The H atoms of the water molecules were located in Fourier difference maps and allowed to ride on their parent atoms with *U*_iso_(H) = 1.5*U*_eq_(O)

### Crystal data

**Table:**

[Cu_4_(C_7_H_4_O_3_)_2_(OH)_2_(C_10_H_8_N_2_)_4_](NO_3_)_2_·4H_2_O	*Z* = 1
*M**_r_* = 1381.20	*F*(000) = 704
Triclinic, *P*1	*D*_x_ = 1.715 Mg m^−^^3^
Hall symbol: -P 1	Mo *K*α radiation, λ = 0.71073 Å
*a* = 10.280 (2) Å	Cell parameters from 3709 reflections
*b* = 11.777 (2) Å	θ = 2.0–27.9°
*c* = 12.276 (3) Å	µ = 1.66 mm^−^^1^
α = 113.66 (3)°	*T* = 113 K
β = 95.19 (3)°	Platelet, blue
γ = 96.58 (3)°	0.22 × 0.06 × 0.02 mm
*V* = 1337.0 (5) Å^3^	

### Data collection

**Table:**

Rigaku Saturn70 diffractometer	6339 independent reflections
Radiation source: rotating anode	4704 reflections with *I* > 2σ(*I*)
confocal	*R*_int_ = 0.049
Detector resolution: 28.5714 pixels mm^-1^	θ_max_ = 27.9°, θ_min_ = 1.8°
ω scans	*h* = −13→13
Absorption correction: multi-scan (*CrystalClear*; Rigaku, 2005)	*k* = −15→15
*T*_min_ = 0.870, *T*_max_ = 1.000	*l* = −16→16
17013 measured reflections	

### Refinement

**Table:**

Refinement on *F*^2^	Primary atom site location: structure-invariant direct methods
Least-squares matrix: full	Secondary atom site location: difference Fourier map
*R*[*F*^2^ > 2σ(*F*^2^)] = 0.040	Hydrogen site location: inferred from neighbouring sites
*wR*(*F*^2^) = 0.086	H-atom parameters constrained
*S* = 1.04	*w* = 1/[σ^2^(*F*_o_^2^) + (0.0365*P*)^2^] where *P* = (*F*_o_^2^ + 2*F*_c_^2^)/3
6339 reflections	(Δ/σ)_max_ = 0.001
388 parameters	Δρ_max_ = 0.81 e Å^−^^3^
0 restraints	Δρ_min_ = −0.55 e Å^−^^3^

### Special details

**Table:**

Geometry. All esds (except the esd in the dihedral angle between two l.s. planes) are estimated using the full covariance matrix. The cell esds are taken into account individually in the estimation of esds in distances, angles and torsion angles; correlations between esds in cell parameters are only used when they are defined by crystal symmetry. An approximate (isotropic) treatment of cell esds is used for estimating esds involving l.s. planes.
Refinement. Refinement of *F*^2^ against ALL reflections. The weighted *R*-factor *wR* and goodness of fit *S* are based on *F*^2^, conventional *R*-factors *R* are based on *F*, with *F* set to zero for negative *F*^2^. The threshold expression of *F*^2^ > σ(*F*^2^) is used only for calculating *R*-factors(gt) *etc*. and is not relevant to the choice of reflections for refinement. *R*-factors based on *F*^2^ are statistically about twice as large as those based on *F*, and *R*- factors based on ALL data will be even larger.

### Fractional atomic coordinates and isotropic or equivalent isotropic displacement parameters (Å^2^)

**Table:**

	*x*	*y*	*z*	*U*_iso_*/*U*_eq_	
Cu1	0.51741 (3)	0.52708 (3)	0.39192 (3)	0.01034 (9)	
Cu2	0.22148 (3)	0.45741 (3)	0.39624 (3)	0.01153 (9)	
O1	0.18797 (17)	0.37760 (17)	0.22317 (15)	0.0141 (4)	
O2	0.15228 (19)	0.38000 (18)	0.04362 (15)	0.0213 (4)	
O3	0.32811 (16)	0.60131 (16)	0.39640 (15)	0.0117 (4)	
O4	0.42106 (16)	0.40125 (16)	0.43430 (14)	0.0111 (4)	
H4W	0.4216	0.3340	0.4009	0.017*	
N1	0.6105 (2)	0.6598 (2)	0.35125 (18)	0.0118 (4)	
N2	0.5192 (2)	0.4242 (2)	0.21681 (18)	0.0124 (5)	
N3	0.1940 (2)	0.5494 (2)	0.56784 (18)	0.0129 (5)	
N4	0.11500 (19)	0.3170 (2)	0.41558 (19)	0.0125 (5)	
C1	0.1809 (2)	0.4365 (3)	0.1550 (2)	0.0137 (5)	
C2	0.2038 (2)	0.5778 (3)	0.2097 (2)	0.0133 (5)	
C3	0.1470 (3)	0.6394 (3)	0.1445 (2)	0.0186 (6)	
H3	0.0955	0.5918	0.0699	0.022*	
C4	0.1661 (3)	0.7685 (3)	0.1887 (3)	0.0210 (6)	
H4	0.1250	0.8074	0.1457	0.025*	
C5	0.2466 (3)	0.8402 (3)	0.2976 (2)	0.0191 (6)	
H5	0.2630	0.9272	0.3257	0.023*	
C6	0.3031 (2)	0.7829 (3)	0.3652 (2)	0.0149 (5)	
H6	0.3570	0.8319	0.4382	0.018*	
C7	0.2792 (2)	0.6517 (2)	0.3240 (2)	0.0111 (5)	
C8	0.6352 (2)	0.7830 (3)	0.4223 (2)	0.0155 (6)	
H8	0.6160	0.8105	0.5005	0.019*	
C9	0.6884 (3)	0.8708 (3)	0.3830 (2)	0.0183 (6)	
H9	0.7009	0.9563	0.4329	0.022*	
C10	0.7227 (3)	0.8296 (3)	0.2684 (2)	0.0206 (6)	
H10	0.7614	0.8867	0.2411	0.025*	
C11	0.6983 (3)	0.7019 (3)	0.1951 (2)	0.0185 (6)	
H11	0.7221	0.6719	0.1184	0.022*	
C12	0.6383 (2)	0.6195 (3)	0.2374 (2)	0.0138 (5)	
C13	0.4658 (3)	0.3033 (3)	0.1531 (2)	0.0157 (6)	
H13	0.4132	0.2630	0.1890	0.019*	
C14	0.4855 (3)	0.2356 (3)	0.0354 (2)	0.0187 (6)	
H14	0.4479	0.1513	−0.0064	0.022*	
C15	0.5623 (3)	0.2961 (3)	−0.0183 (2)	0.0203 (6)	
H15	0.5789	0.2524	−0.0963	0.024*	
C16	0.6144 (3)	0.4225 (3)	0.0452 (2)	0.0178 (6)	
H16	0.6641	0.4652	0.0095	0.021*	
C17	0.5915 (2)	0.4845 (3)	0.1626 (2)	0.0126 (5)	
C18	0.2375 (2)	0.6718 (2)	0.6373 (2)	0.0145 (5)	
H18	0.2852	0.7200	0.6054	0.017*	
C19	0.2137 (3)	0.7287 (3)	0.7552 (2)	0.0192 (6)	
H19	0.2441	0.8139	0.8017	0.023*	
C20	0.1436 (3)	0.6557 (3)	0.8020 (2)	0.0200 (6)	
H20	0.1261	0.6915	0.8807	0.024*	
C21	0.0998 (2)	0.5291 (3)	0.7310 (2)	0.0168 (6)	
H21	0.0539	0.4789	0.7618	0.020*	
C22	0.1252 (2)	0.4782 (3)	0.6133 (2)	0.0137 (6)	
C23	0.0808 (3)	0.1999 (3)	0.3308 (2)	0.0177 (6)	
H23	0.1046	0.1817	0.2550	0.021*	
C24	0.0113 (3)	0.1040 (3)	0.3514 (2)	0.0193 (6)	
H24	−0.0108	0.0228	0.2912	0.023*	
C25	−0.0243 (3)	0.1336 (3)	0.4651 (3)	0.0215 (6)	
H25	−0.0719	0.0717	0.4815	0.026*	
C26	0.0107 (2)	0.2547 (3)	0.5538 (2)	0.0176 (6)	
H26	−0.0125	0.2749	0.6301	0.021*	
C27	0.0809 (2)	0.3453 (3)	0.5271 (2)	0.0137 (5)	
O5	0.5540 (2)	0.0871 (2)	0.3318 (2)	0.0436 (6)	
O6	0.4419 (2)	−0.0024 (2)	0.15320 (19)	0.0460 (7)	
O7	0.3639 (2)	0.13765 (19)	0.29450 (18)	0.0261 (5)	
N5	0.4554 (2)	0.0740 (2)	0.2603 (2)	0.0219 (5)	
O8	0.1781 (2)	0.9602 (2)	0.01777 (19)	0.0361 (6)	
H1W	0.2414	0.9788	0.0663	0.054*	
H2W	0.1077	0.9446	0.0479	0.054*	
O9	0.0737 (2)	0.1196 (2)	0.93420 (18)	0.0336 (5)	
H3W	0.1170	0.0894	0.9614	0.050*	
H5W	0.0904	0.1926	0.9720	0.050*	

### Atomic displacement parameters (Å^2^)

**Table:**

	*U*^11^	*U*^22^	*U*^33^	*U*^12^	*U*^13^	*U*^23^
Cu1	0.01258 (16)	0.01008 (17)	0.00903 (15)	0.00210 (13)	0.00294 (12)	0.00431 (13)
Cu2	0.01248 (17)	0.01164 (17)	0.01137 (16)	0.00126 (13)	0.00235 (12)	0.00577 (13)
O1	0.0184 (10)	0.0127 (9)	0.0116 (9)	0.0013 (8)	0.0023 (7)	0.0058 (8)
O2	0.0302 (11)	0.0198 (11)	0.0104 (9)	0.0007 (9)	0.0018 (8)	0.0041 (8)
O3	0.0091 (9)	0.0131 (9)	0.0155 (9)	0.0020 (7)	0.0012 (7)	0.0087 (8)
O4	0.0144 (9)	0.0090 (9)	0.0105 (9)	0.0034 (7)	0.0024 (7)	0.0040 (7)
N1	0.0123 (11)	0.0132 (11)	0.0115 (10)	0.0048 (9)	0.0038 (9)	0.0057 (9)
N2	0.0145 (11)	0.0143 (11)	0.0100 (10)	0.0053 (9)	0.0033 (9)	0.0056 (9)
N3	0.0126 (11)	0.0123 (11)	0.0135 (11)	0.0017 (9)	0.0014 (9)	0.0053 (9)
N4	0.0093 (11)	0.0156 (12)	0.0151 (11)	0.0034 (9)	0.0035 (9)	0.0081 (10)
C1	0.0106 (13)	0.0163 (14)	0.0134 (13)	0.0028 (11)	0.0042 (11)	0.0048 (11)
C2	0.0127 (13)	0.0175 (14)	0.0145 (13)	0.0051 (11)	0.0067 (11)	0.0098 (11)
C3	0.0166 (14)	0.0275 (17)	0.0174 (14)	0.0024 (12)	0.0030 (11)	0.0153 (13)
C4	0.0193 (15)	0.0261 (16)	0.0275 (16)	0.0084 (13)	0.0063 (13)	0.0195 (14)
C5	0.0208 (15)	0.0166 (15)	0.0270 (15)	0.0060 (12)	0.0113 (12)	0.0138 (13)
C6	0.0118 (13)	0.0174 (14)	0.0152 (13)	0.0020 (11)	0.0023 (11)	0.0065 (11)
C7	0.0085 (12)	0.0134 (13)	0.0146 (13)	0.0052 (10)	0.0056 (10)	0.0073 (11)
C8	0.0157 (13)	0.0153 (14)	0.0155 (13)	0.0031 (11)	0.0053 (11)	0.0057 (11)
C9	0.0183 (14)	0.0142 (14)	0.0187 (14)	−0.0009 (12)	−0.0012 (11)	0.0050 (12)
C10	0.0210 (15)	0.0212 (16)	0.0234 (15)	−0.0024 (12)	0.0049 (12)	0.0145 (13)
C11	0.0173 (14)	0.0252 (16)	0.0161 (13)	0.0012 (12)	0.0036 (11)	0.0121 (13)
C12	0.0112 (13)	0.0186 (14)	0.0140 (13)	0.0037 (11)	0.0029 (10)	0.0086 (12)
C13	0.0168 (14)	0.0155 (14)	0.0148 (13)	0.0036 (11)	0.0048 (11)	0.0056 (11)
C14	0.0208 (15)	0.0166 (15)	0.0148 (13)	0.0061 (12)	0.0027 (11)	0.0019 (12)
C15	0.0210 (15)	0.0260 (17)	0.0129 (13)	0.0105 (13)	0.0040 (12)	0.0050 (12)
C16	0.0205 (14)	0.0238 (16)	0.0110 (13)	0.0073 (13)	0.0040 (11)	0.0077 (12)
C17	0.0093 (12)	0.0179 (14)	0.0133 (12)	0.0060 (11)	0.0019 (10)	0.0084 (11)
C18	0.0149 (13)	0.0122 (13)	0.0162 (13)	0.0035 (11)	0.0036 (11)	0.0051 (11)
C19	0.0180 (14)	0.0184 (15)	0.0178 (14)	0.0070 (12)	−0.0001 (12)	0.0038 (12)
C20	0.0165 (14)	0.0278 (17)	0.0147 (13)	0.0104 (13)	0.0044 (11)	0.0055 (13)
C21	0.0124 (13)	0.0246 (16)	0.0188 (14)	0.0087 (12)	0.0063 (11)	0.0121 (12)
C22	0.0066 (12)	0.0213 (15)	0.0192 (13)	0.0059 (11)	0.0031 (11)	0.0133 (12)
C23	0.0171 (14)	0.0164 (14)	0.0179 (14)	0.0027 (12)	0.0019 (11)	0.0056 (12)
C24	0.0159 (14)	0.0141 (14)	0.0256 (15)	0.0015 (11)	0.0025 (12)	0.0061 (12)
C25	0.0160 (14)	0.0193 (15)	0.0354 (17)	0.0019 (12)	0.0074 (13)	0.0173 (14)
C26	0.0123 (13)	0.0224 (15)	0.0229 (15)	0.0048 (12)	0.0062 (11)	0.0132 (13)
C27	0.0100 (13)	0.0171 (14)	0.0176 (13)	0.0051 (11)	0.0014 (11)	0.0102 (12)
O5	0.0376 (14)	0.0415 (16)	0.0478 (15)	−0.0024 (12)	−0.0136 (12)	0.0212 (13)
O6	0.0614 (17)	0.0378 (15)	0.0241 (12)	0.0200 (13)	0.0100 (12)	−0.0061 (11)
O7	0.0354 (12)	0.0184 (11)	0.0284 (11)	0.0106 (10)	0.0153 (10)	0.0099 (9)
N5	0.0317 (14)	0.0129 (12)	0.0208 (13)	0.0012 (11)	0.0040 (11)	0.0073 (11)
O8	0.0337 (13)	0.0347 (14)	0.0369 (13)	0.0057 (11)	−0.0046 (10)	0.0138 (11)
O9	0.0485 (14)	0.0204 (12)	0.0291 (12)	0.0020 (10)	−0.0022 (10)	0.0101 (10)

### Geometric parameters (Å, °)

**Table:**

Cu1—O4	1.9554 (18)	C9—H9	0.9300
Cu1—O4^i^	1.9626 (18)	C10—C11	1.386 (4)
Cu1—N1	1.995 (2)	C10—H10	0.9300
Cu1—N2	2.003 (2)	C11—C12	1.384 (4)
Cu1—O3	2.2191 (17)	C11—H11	0.9300
Cu1—Cu1^i^	3.0090 (9)	C12—C17	1.475 (4)
Cu2—O3	1.9092 (18)	C13—C14	1.390 (3)
Cu2—O1	1.9253 (18)	C13—H13	0.9300
Cu2—N4	1.984 (2)	C14—C15	1.381 (4)
Cu2—N3	2.009 (2)	C14—H14	0.9300
Cu2—O4	2.2914 (17)	C15—C16	1.386 (4)
O1—C1	1.286 (3)	C15—H15	0.9300
O2—C1	1.246 (3)	C16—C17	1.386 (3)
O3—C7	1.344 (3)	C16—H16	0.9300
O4—Cu1^i^	1.9626 (18)	C18—C19	1.389 (3)
O4—H4W	0.7321	C18—H18	0.9300
N1—C8	1.338 (3)	C19—C20	1.385 (4)
N1—C12	1.354 (3)	C19—H19	0.9300
N2—C13	1.338 (3)	C20—C21	1.384 (4)
N2—C17	1.359 (3)	C20—H20	0.9300
N3—C18	1.343 (3)	C21—C22	1.388 (3)
N3—C22	1.350 (3)	C21—H21	0.9300
N4—C23	1.334 (3)	C22—C27	1.483 (4)
N4—C27	1.363 (3)	C23—C24	1.387 (4)
C1—C2	1.503 (4)	C23—H23	0.9300
C2—C3	1.410 (3)	C24—C25	1.391 (4)
C2—C7	1.413 (4)	C24—H24	0.9300
C3—C4	1.376 (4)	C25—C26	1.384 (4)
C3—H3	0.9300	C25—H25	0.9300
C4—C5	1.385 (4)	C26—C27	1.382 (4)
C4—H4	0.9300	C26—H26	0.9300
C5—C6	1.390 (4)	O5—N5	1.231 (3)
C5—H5	0.9300	O6—N5	1.243 (3)
C6—C7	1.403 (3)	O7—N5	1.266 (3)
C6—H6	0.9300	O8—H1W	0.7861
C8—C9	1.386 (4)	O8—H2W	0.8758
C8—H8	0.9300	O9—H3W	0.7356
C9—C10	1.386 (4)	O9—H5W	0.7848
			
O4—Cu1—O4^i^	79.65 (8)	C6—C7—C2	119.3 (2)
O4—Cu1—N1	177.76 (7)	N1—C8—C9	122.1 (2)
O4^i^—Cu1—N1	99.87 (9)	N1—C8—H8	119.0
O4—Cu1—N2	100.21 (9)	C9—C8—H8	119.0
O4^i^—Cu1—N2	157.28 (7)	C10—C9—C8	119.1 (3)
N1—Cu1—N2	81.09 (9)	C10—C9—H9	120.5
O4—Cu1—O3	85.03 (7)	C8—C9—H9	120.5
O4^i^—Cu1—O3	98.53 (7)	C11—C10—C9	118.8 (3)
N1—Cu1—O3	92.88 (7)	C11—C10—H10	120.6
N2—Cu1—O3	104.11 (8)	C9—C10—H10	120.6
O4—Cu1—Cu1^i^	39.91 (5)	C12—C11—C10	119.3 (2)
O4^i^—Cu1—Cu1^i^	39.74 (5)	C12—C11—H11	120.4
N1—Cu1—Cu1^i^	139.57 (7)	C10—C11—H11	120.4
N2—Cu1—Cu1^i^	135.79 (7)	N1—C12—C11	121.5 (3)
O3—Cu1—Cu1^i^	92.32 (5)	N1—C12—C17	114.3 (2)
O3—Cu2—O1	92.07 (8)	C11—C12—C17	124.0 (2)
O3—Cu2—N4	173.73 (8)	N2—C13—C14	122.7 (3)
O1—Cu2—N4	94.20 (9)	N2—C13—H13	118.7
O3—Cu2—N3	92.89 (9)	C14—C13—H13	118.7
O1—Cu2—N3	160.86 (8)	C15—C14—C13	118.5 (3)
N4—Cu2—N3	81.20 (9)	C15—C14—H14	120.7
O3—Cu2—O4	84.10 (7)	C13—C14—H14	120.7
O1—Cu2—O4	101.26 (7)	C14—C15—C16	119.4 (2)
N4—Cu2—O4	94.55 (7)	C14—C15—H15	120.3
N3—Cu2—O4	97.63 (8)	C16—C15—H15	120.3
C1—O1—Cu2	124.61 (16)	C15—C16—C17	119.2 (3)
C7—O3—Cu2	117.08 (15)	C15—C16—H16	120.4
C7—O3—Cu1	126.56 (14)	C17—C16—H16	120.4
Cu2—O3—Cu1	95.78 (7)	N2—C17—C16	121.5 (3)
Cu1—O4—Cu1^i^	100.35 (8)	N2—C17—C12	114.5 (2)
Cu1—O4—Cu2	92.23 (7)	C16—C17—C12	123.9 (2)
Cu1^i^—O4—Cu2	110.45 (8)	N3—C18—C19	122.1 (3)
Cu1—O4—H4W	121.6	N3—C18—H18	119.0
Cu1^i^—O4—H4W	116.7	C19—C18—H18	119.0
Cu2—O4—H4W	112.6	C20—C19—C18	118.4 (3)
C8—N1—C12	119.1 (2)	C20—C19—H19	120.8
C8—N1—Cu1	125.48 (17)	C18—C19—H19	120.8
C12—N1—Cu1	115.12 (18)	C21—C20—C19	119.6 (3)
C13—N2—C17	118.6 (2)	C21—C20—H20	120.2
C13—N2—Cu1	126.92 (18)	C19—C20—H20	120.2
C17—N2—Cu1	114.28 (18)	C20—C21—C22	119.2 (3)
C18—N3—C22	119.5 (2)	C20—C21—H21	120.4
C18—N3—Cu2	125.57 (18)	C22—C21—H21	120.4
C22—N3—Cu2	114.89 (18)	N3—C22—C21	121.1 (3)
C23—N4—C27	119.5 (2)	N3—C22—C27	114.3 (2)
C23—N4—Cu2	125.18 (18)	C21—C22—C27	124.5 (2)
C27—N4—Cu2	115.27 (18)	N4—C23—C24	122.6 (2)
O2—C1—O1	121.9 (2)	N4—C23—H23	118.7
O2—C1—C2	118.1 (2)	C24—C23—H23	118.7
O1—C1—C2	119.9 (2)	C23—C24—C25	117.7 (3)
C3—C2—C7	118.5 (2)	C23—C24—H24	121.1
C3—C2—C1	118.6 (2)	C25—C24—H24	121.1
C7—C2—C1	122.9 (2)	C26—C25—C24	120.3 (3)
C4—C3—C2	121.4 (3)	C26—C25—H25	119.9
C4—C3—H3	119.3	C24—C25—H25	119.9
C2—C3—H3	119.3	C27—C26—C25	118.8 (2)
C3—C4—C5	119.7 (3)	C27—C26—H26	120.6
C3—C4—H4	120.1	C25—C26—H26	120.6
C5—C4—H4	120.1	N4—C27—C26	121.1 (3)
C4—C5—C6	120.5 (3)	N4—C27—C22	114.2 (2)
C4—C5—H5	119.7	C26—C27—C22	124.6 (2)
C6—C5—H5	119.7	O5—N5—O6	121.4 (3)
C5—C6—C7	120.4 (2)	O5—N5—O7	120.6 (3)
C5—C6—H6	119.8	O6—N5—O7	118.0 (2)
C7—C6—H6	119.8	H1W—O8—H2W	109.5
O3—C7—C6	118.2 (2)	H3W—O9—H5W	108.6
O3—C7—C2	122.5 (2)		

Symmetry codes: (i) −*x*+1, −*y*+1, −*z*+1.

### Hydrogen-bond geometry (Å, °)

**Table:**

*D*—H···*A*	*D*—H	H···*A*	*D*···*A*	*D*—H···*A*
O8—H1W···O6^ii^	0.79	2.18	2.939 (3)	163
O8—H2W···O9^iii^	0.88	2.00	2.845 (3)	163
O9—H3W···O8^iv^	0.74	2.04	2.745 (3)	160
O4—H4W···O7	0.73	2.13	2.838 (3)	164
O9—H5W···O2^v^	0.78	2.02	2.791 (3)	169

Symmetry codes: (ii) *x*, *y*+1, *z*; (iii) −*x*, −*y*+1, −*z*+1; (iv) *x*, *y*−1, *z*+1;  (v) *x*, *y*, *z*+1.
